# Myocardial ASL perfusion reserve test detects ischemic segments in initial cohort of 10 patients with angiographic CAD

**DOI:** 10.1186/1532-429X-13-S1-P110

**Published:** 2011-02-02

**Authors:** Zungho Zun, Terrence Jao, Ning Smith, Padmini Varadarajan, Ramdas G Pai, Eric C Wong, Krishna S Nayak

**Affiliations:** 1 University of Southern California, Los Angeles, CA, USA; 2 Kaiser Permanente Southern California, Pasadena, CA, USA; 3 Loma Linda University Medical Center, Loma Linda, CA, USA; 4 University of California, San Diego, La Jolla, CA, USA

## Objective

This study sought to demonstrate the potential for myocardial arterial spin labeling (ASL) to identify the ischemic myocardial segments due to stenosis in coronary arteries as detected by X-ray angiography.

## Background

Myocardial ASL is a technique for the assessment of myocardial blood flow (MBF) without contrast agents. It can be safely applied to patients with end-stage renal disease who are not candidates for first-pass imaging with contrast agents. Myocardial ASL perfusion imaging performed at rest and during adenosine stress provides perfusion reserve (MBF_stress_/MBF_rest_), which is a common indicator for the severity of coronary artery disease. In healthy myocardium, perfusion reserve is known to be approximately four [1].

## Methods

Twenty nine patients were recruited from those scheduled for routine cardiac MR (CMR) and X-ray angiography. Myocardial ASL measurements were obtained from a single mid short-axis slice at rest and during adenosine infusion (dosage: 0.14 mg/kg/min) on a GE Signa 3T scanner. The ASL sequence was composed of flow-sensitive alternating inversion recovery (FAIR) tagging and balanced steady-state free precession (SSFP) imaging [2]. Perfusion reserve maps were generated in a standard short-axis view illustration by convolution with a Gaussian filter and resampling onto a polar coordinate [3].

## Results

**Figure 1:**
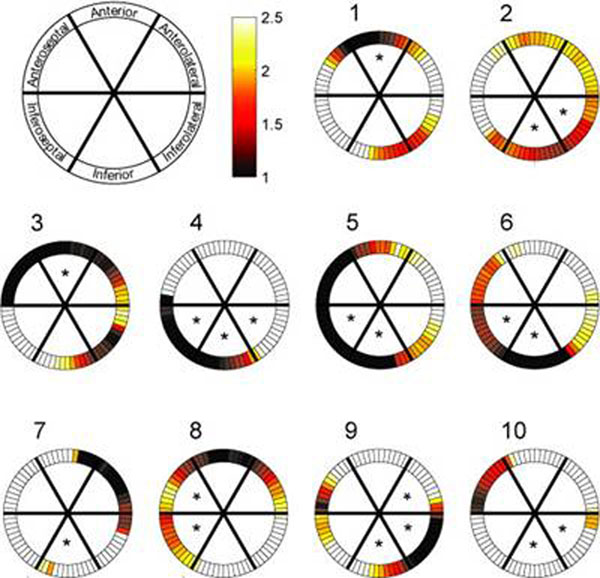
Perfusion reserve maps acquired using myocardial ASL in patients 1-10. Asterisks denote the most ischemic segments identified based on X-ray angiography.

Ten of the twenty-nine patients were found to have significant stenosis on X-ray angiography. Table 1 contains the most ischemic myocardial segments in these ten patients as identified by two cardiologists using either X-ray angiogram or ASL perfusion reserve map independently. Based on McNemar’s test with Bonferroni correction, there was no significant difference between X-ray and ASL MRI in identifying ischemia in all six myocardial segments (p = 1.0000, 0.6170, 0.4795, 0.1336, 0.4795, and 0.4795). Figure 1 contains perfusion reserve maps acquired using myocardial ASL in these patients. The average standard deviation of physiological noise was 0.22 ml/g/min at rest and 0.42 ml/g/min during stress [2].

**Table 1:** Most ischemic myocardial segments identified by X-ray angiograms and by ASL perfusion reserve maps

Pts #	X-ray angiography		ASL MRI
	Worst lesion on angiogram	Ischemic myocardial segments	Ischemic myocardial segments
1	Proximal LAD 100%	Anterior	Anterior
2	RCA 100%	Inferior, inferolateral	Inferolateral
3	LAD 90%	Anterior	Anterior, anteroseptal
4	LCS 90% (PDA)	Inferoseptal, inferior, inferolateral	Inferoseptal, inferior
5	RCA (100%)	Inferoseptal, inferior	Anteroseptal, inferoseptal, inferior
6	RCA (100%)	Inferoseptal, inferior	Inferior
7	Distal RCA 80%	Inferior	Anterolateral
8	Stent to LAD and RCA – now open	Anteroseptal, inferoseptal	Anterior
9	LCX 100%, RCA 100%	Inferior, inferolateral, anterolateral	Inferolateral
10	RCA 100%	Inferior, inferolateral	Anteroseptal

## Conclusion

There was visual agreement (except patients 7, 8, and 10) and no statistically significant difference between ischemic myocardial segments identified by ASL perfusion reserve maps and by X-ray angiograms. This suggests that myocardial ASL with vasodilation may have a potential to identify ischemic myocardial segments in patients with stenosis.
